# The Atrial Fibrillation Better Care pathway for managing atrial fibrillation: a review

**DOI:** 10.1093/europace/euab092

**Published:** 2021-06-14

**Authors:** David Stevens, Stephanie L Harrison, Ruwanthi Kolamunnage-Dona, Gregory Y H Lip, Deirdre A Lane

**Affiliations:** 1 Liverpool Centre for Cardiovascular Science, University of Liverpool, Liverpool Heart & Chest Hospital, 6 West Derby Street, Liverpool L7 8TX, UK; 2 Cardiovascular and Metabolic Medicine, Institute of Life Course and Medical Sciences, University of Liverpool, Liverpool, UK; 3 Department of Health Data Science, Institute of Population Health, University of Liverpool, Liverpool, UK; 4 Department of Clinical Medicine, Aalborg University, Aalborg, Denmark

**Keywords:** ABC pathway, Integrated care, Atrial fibrillation, Management, Patients, Review

## Abstract

The 2020 European Society of Cardiology guidelines endorse the Atrial Fibrillation Better Care (ABC) pathway as a structured approach for the management of atrial fibrillation (AF), addressing three principal elements: ‘A’ – avoid stroke (with oral anticoagulation), ‘B’ – patient-focused better symptom management, and ‘C’ – cardiovascular and comorbidity risk factor reduction and management. This review summarizes the definitions used for the ABC criteria in different studies and the impact of adherence/non-adherence on clinical outcomes, from 12 studies on seven different cohorts. All studies consistently showed statistically significant reductions in the risk of stroke, myocardial infarction, and mortality among those with ABC pathway adherent treatment. The ABC pathway provides a simple decision-making framework to enable consistent equitable care from clinicians in primary and secondary/tertiary care. Further research examining the impact of ABC pathway implementation in prospective cohorts utilizing consistent inclusion criteria and definitions of ‘A’, ‘B’, and ‘C’ adherent care is warranted.

## Introduction

Atrial fibrillation (AF) is associated with a five-fold increase in the risk of stroke^1^ and a higher risk of cardiovascular and all-cause mortality.^2^ Current European Society of Cardiology (ESC) guidelines on AF management advocate the use of oral anticoagulants (OACs) to reduce stroke risk in patients with a CHA_2_DS_2_-VASc risk score of ≥1 for men and ≥2 for women.^3^

More recently, there has been a move towards recommending an integrated care approach to AF management.^3–8^ Three studies examining integrated care for the management of AF^6–8^ were analysed in a meta-analysis, which showed a significant reduction in the risk of both mortality and hospitalization^9^; however, this systematic review showed inconsistency in the populations recruited and the care provided between the studies.

In 2017, the Atrial Fibrillation Better Care (ABC) pathway was proposed as an integrated, structured approach to AF management,^10^ addressing three main components: ‘A’ refers to ‘avoid stroke’, by offering stroke prevention with appropriate OAC to patients with a CHA_2_DS_2_-VASc score of ≥1 for men and ≥2 for women.^1^. ‘B’ refers to ‘better symptom management’ and involves a patient and symptom-focused approach to decisions on managing heart rate or rhythm. ‘C’ refers to ‘cardiovascular and comorbidity risk reduction’, comprising the management of risk factors for other cardiovascular outcomes.

Several studies^12–23^ have examined the impact of adherence/non-adherence to the ABC pathway. This review summarizes the definitions used for the ABC criteria in different datasets and evaluates the impact of adherence/non-adherence on clinical outcomes.

## Methods

### Literature search

Medline Ovid was searched from inception to 1 December 2020, using the following terms in the title or abstract of the article: ABC or ‘Atrial Fibrillation Better Care’, pathway, and atrial fibrillation. Additionally, studies were examined based on references cited in identified sources and communication with experts in the field.

### Study selection

Papers were included if they defined criteria for ABC pathway adherence in an AF cohort. There were no restrictions based on study design. To be included, studies needed to compare groups of patients who were either ABC adherent or non-ABC adherent or which had an intervention that aimed to improve ABC adherence in one arm of a randomized clinical trial. Reviews and guidelines with no data were excluded. The first author screened the available titles and abstracts, and papers which were potentials for inclusion were discussed and agreed with other authors.

#### Data extraction and synthesis

Data extracted from relevant publications included: first author, year of publication, number of participants, the proportion of males and females, mean [standard deviation (SD)]/median [inter-quartile range (IQR)] age, length of the follow-up period, criteria used for ABC adherence definitions, sample selection criteria, disease outcomes reported, the number of events in ABC adherent and non-ABC adherent groups, and the covariates adjusted for. The first author completed the data extraction, and other authors were consulted to resolve any queries. Following extraction, these data were summarized in tables. The variation in definitions and criteria included to define A, B and C crtieria precluded any attempts to combine the results of individual studies in a meta-analysis.

## Results

The searches for this review returned 19 studies and after reviewing the titles and abstracts, 12 studies^12–23^ were reviewed as full-text and included. Reasons for exclusion included: reviews (*n* = 2), guidelines (*n* = 2), ABC criteria not defined (*n* = 1), wrong population and no reference to ABC pathway (*n* = 1), and wrong outcomes (i.e. costs) (*n* = 1). The 12 included studies used data from seven different datasets. Three datasets were prospectively collected,^12^^,^^15–18^ two were retrospective *post hoc* analyses of prospectively collected data^19^^,^^21^^,^^22^ and two were registries or electronic health records.^13^^,^^14^^,^^20^^,^^23^ Characteristics of the included studies are provided in *Table 1*. Studies used data from around the world: South Korea (*n* = 3),^13^^,^^14^^,^^23^ China (*n* = 2),^17^^,^^18^ the Middle East (*n* = 2),^15^^,^^16^ Italy (*n* = 1),^19^ Europe (*n* = 1),^20^ the USA and Canada (*n* = 2),^21^^,^^22^ and the Balkans (*n* = 1).^12^

**Table 1:** Summary of the characteristics of the included studies

First author (year), country	Study cohort	Cohort description	Selection criteria	Length of follow-up, mean ± SD or median (IQR)	Outcomes
Prospective					
Domek (2020), Middle East^15^	Gulf Survey of Atrial Fibrillation Events (SAFE) Registry	Consecutive patients admitted to ED in 23 hospitals in 6 Middle East countries independently from the primary reason for admission, 603,^a^ 63.42 ± 11.75,^b^ 315 (52.2%),^c^ not reported,^d^ 3.69 ± 1.58,^e^ 1.56 ± 1.07^f^	*Inclusion criteria:* ≥ 18 years old, >30 s AF on 12-lead resting ECG, diabetes	12 months	*Primary:* ACM, composite: stroke/systemic embolism, ACM, CV hospitalization
Gumprecht (2020), Middle East^16^	Gulf Survey of Atrial Fibrillation Events (SAFE) Registry	Consecutive patients admitted to ED in 23 hospitals in 6 Middle East countries independently from the primary reason for admission, 2021,^a^ 56.74 ± 16.47,^b^ 968 (47.9%),^c^ not reported,^d^ 2.34 ± 1.78,^e^ 1.13 ± 1.065^f^	*Inclusion criteria:* ≥18 years old, >30 s AF on 12-lead resting electrocardiogram. *Exclusion criteria:* insufficient data for calculating CHA_2_DS_2_-VASc score	1 year	*Primary:* ACM, composite of ischaemic stroke or systemic embolism/all-cause mortality and CV hospitalization
Guo (2020) 1 year, China^18^	mAFA II trial	2 arm cluster-RCT. Clusters were 40 Chinese hospitals, 3324,^a^ mAFA: 67.0 ± 15.0 UC: 70.0 ± 12.0,^b^ mAFA: 625 (38.0%) UC: 637 (38.0%),^c^ not reported,^d^ mAFA: 3 (2–4) UC: 3 (2–4),^e^ mAFA: 1 (1–2) UC: 1 (1–2)^f^	*Inclusion criteria:* ≥18 years old, AF confirmed by ECG or 24-h Holter, CHA_2_DS_2_-VASc ≥2. *Exclusion criteria:* mechanical prosthetic value or moderate/severe mitral stenosis, unable to provide informed consent, unable to be followed up for 1 year for any reason	12 months	*Primary:* composite: stroke/thromboembolism, ACM, and re-hospitalization
Guo (2020) extension, China^17^	mAFA II trial	2 arm cluster-RCT. Clusters were 40 Chinese hospitals, 2473,^a^ mAFA: 67.8 ± 15.4 UC: 70.1 ± 12.0,^b^ mAFA: 430 (34.1%) UC: 511 (42.1%),^c^ not reported,^d^ mAFA: 3 (2–4) UC: 3 (2–4),^e^ mAFA: 2 (1–3) UC: 2 (1–3)^f^	*Inclusion criteria:* ≥18 years old, AF confirmed by ECG or 24-h Holter, CHA_2_DS_2_-VASc ≥2, Over 1 year of follow-up. *Exclusion criteria:* mechanical prosthetic value or moderate/severe mitral stenosis, unable to provide informed consent	mAFA: 687 ± 191; 701 (489–841) days, usual care: 514 ± 167; 546 (394–632) days	*Primary:* composite: stroke/thromboembolism, ACM, and re-hospitalization. *Secondary:* ischaemic stroke, other thromboembolism, intracranial bleeding, extracranial bleeding, recurrent AF or AF symptom, heart failure, ACM
Koziel (2020), Balkans^12^	BALKAN-AF survey	Consecutive patients managed in hospitals and outpatient settings; 8 Balkan countries; 49 centres; 14-week observational survey recorded prospectively, 2712,^a^ ABC: 49 (41, 57) non-ABC: 64 (55, 71),^b^ ABC: 485 (47.9%) non-ABC: 557 (42.9%),^c^ not reported,^d^ ABC: 3.4 ± 1.8 non-ABC: 3.4 ± 1.9,^e^ ABC: 1.94 ± 1.2 non-ABC: 1.99 ± 1.2^f^	*Inclusion criteria:* ≥18 years old. *Exclusion criteria:* prosthetic mechanical heart valves, moderate or severe mitral valve stenosis or any significant heart valve disease with indications for surgical treatment	None	*Primary:* ABC adherence
Retrospective—*post hoc*					
Proietti (2018, 2020), USA and Canada^21^^,^^22^	AFFIRM	Retrospective analysis of RCT comparing rate vs. rhythm control and OAC.; 200 sites in USA and Canada, 3169,^a^ 70 (65–76),^b^ 1237 (39.0%),^c^ NR,^d^ 3 (2–4),^e^ not reported^f^	*Inclusion criteria:* on VKA—warfarin, documented AF within last 6 weeks, aged ≥65 years, or <65 years with ≥1 risk factor for stroke, AF episodes in last 6 months totalling ≥6 h, unless cardioversion within 6 h, continuous AF <6 months, unless SR restored and maintained ≥24 h, eligible for rate and rhythm control, eligible for ≥2 AADs (or 2 dose levels of amiodarone) and ≥2 rate-control drugs	3.7 (2.8–4.6)	*Primary:* ACM, composite: stroke/major bleeding/CV mortality, hospitalization. *Secondary:* stroke, major bleeding, CV mortality, CV hospitalization, recurrent hospitalization, total hospitalizations, length of stay for first hospitalization, total length of stay
Pastori (2019), Italy^19^	ATHERO-AF	Single-centre cohort study in Rome, February 2008 to December 2016; Retrospective analysis on prospective observational study, 882,^a^ 73.1 ± 8.5,^b^ 40.8%,^c^ not reported,^d^ 3.50 ± 1.5,^e^ not reported^f^	*Inclusion criteria:* ≥18 years old, AF, all patients on warfarin after risk stratification: CHA_2_DS_2_-VASc for men/women: 0/1—maybe aspirin but no OAC, 1/2 maybe aspirin but preferably OAC, 2+/3+ OAC. *Exclusion criteria:* prosthetic heart valves or severe valvulopathies, severe cognitive impairment, chronic infections (HIV, hepatitis B or C), systemic autoimmune disease, active cancer, liver insufficiency (e.g. cirrhosis)	36.9 (20.0–57.5) months	*Primary:* CV events
Retrospective—Registry or Electronic health records					
Yoon (2019), South Korea^14^	Korea National Health Insurance Service database	National cohort; data from 2005 to 2015; retrospective analysis, 204842,^a^ ABC: 52.9 ± 12.2 non-ABC: 64.9 ± 10.8,^b^ ABC: 10129 (32.0%) non-ABC: 66778 (38.6%),^c^ not reported,^d^ ABC: 0.91 ± 1.39 non-ABC: 2.97 ± 1.80,^e^ not reported^f^	*Inclusion criteria:* adult, non-valvular AF, baseline health check-up data within the year before enrolment, AF outpatient clinic visit during the follow-up period	6.2 ± 3.5 years	*Primary:* ACM, ischaemic stroke, major bleeding, myocardial infarction, composite of other 4 outcomes
Proietti (2020) ESC-EHRA, Europe^20^	ESC-EORP Atrial Fibrillation General Long-Term Registry	Multicentre observational registry held by the ESC and endorsed by the European Heart Rhythm Association (EHRA), 9663,^a^ ABC: 70 (61–76) non-ABC: 69 (61–76),^b^ ABC: 741 (37.1%), non-ABC: 1926 (41.4%),^c^ not reported,^c^ ABC: 2.68 ± 1.57; 3 (2–4) non-ABC: 3.07 ± 1.90; 3 (2–4),^e^ ABC: 1.58 ± 1.12; 2 (1–2) non-ABC: 1.26 ± 0.93; 1 (1–2)^f^	*Inclusion criteria:* ≥ 18 years old, AF documented within 12 months before enrolment based on objective electrocardiographic evaluation	12 months	*Primary:* composite: TE, ACS, CV mortality, CV mortality, ACM, Stroke, Any TE, bleeding events, ICH, any readmission, any AF readmission, any CV readmission, ACS
Yang (2020) dementia, South Korea^23^	Korea National Health Insurance Service database	National cohort; data from 2005 to 2015, 228026,^a^ ABC: 68.8 ± 10.2 non-ABC: 69.7 ± 11.6,^b^ ABC: 18016 (39.2%) non-ABC: 70218 (38.6%),^c^ not reported,^d^ ABC: 0 (0–1) non-ABC: 2 (1–3),^e^ ABC: 0 (0–1) non-ABC: 2 (1–3)^f^	*Inclusion criteria:* ≥18 years old, non-valvular AF, have baseline health check-up data within the year before enrolment. *Exclusion criteria:* patients who had an ischaemic stroke, patients with a history of dementia, patients with an ischaemic stroke during the follow-up period	6.0 (3.3–9.5) years	*Primary:* dementia. *Secondary:* Alzheimer’s disease, vascular dementia
Yang (2020) frailty, South Korea^13^	Korea National Health Insurance Service database	National cohort; data from 2005 to 2015, 262 987,^a^ ABC: 50 (41, 58) non-ABC: 65 (56,72),^b^ ABC: 39.4%, non-ABC: 38.6%,^c^ not reported,^d^ ABC: 0 (0–1), non-ABC: 2 (1–3),^e^ ABC: 0 (0–1), non-ABC: 2 (1–3)^f^	*Inclusion criteria:* ≥18 years old, non-valvular AF, Have baseline health check-up data within the year before enrolment. *Exclusion criteria:* patients who had an ischaemic stroke	5.9 (3.2, 9.4)	*Primary:* ACM, ischaemic stroke, heart failure admission, myocardial infarction, major bleeding, composite of other 5 outcomes

AADs, anti-arrhythmic drugs; ABC, Atrial Fibrillation Better Care; ACM, all-cause mortality; ACS, acute coronary syndrome; AF, atrial fibrillation; CV, cardiovascular; ED, emergency department; EHRA, European Heart Rhythm Association; ESC, European Society of Cardiology; ICH, intra-cranial haemorrhage; mAFA, mobile AF-App; RCT, randomized controlled trial; TE, thromboembolism; UC, usual care.

a *N*.

b Mean ± SD or median (IQR) age.

c *N* (%) female.

d Ethnicity.

e Mean ± SD or median (IQR) CHA_2_DS_2_-VASc score.

f Mean ± SD or median (IQR) HAS-BLED score.

Sample sizes varied from 603 in the Gulf Survey of Atrial Fibrillation Events (SAFE) Registry^15^ to over 260 000 in the Korea National Health Insurance Service database.^13^ Age varied considerably between studies, ranging from 56.7^16^ to 73.1 years.^19^ Two studies had a difference of over 8 years in mean age between ABC adherent and non-ABC adherent patients.^13^^,^^16^ The proportion of women included in each study ranged from 37.5%^14^ to 52.2%.^15^

The follow-up times of six of the studies were relatively short, at only 1–2 years.^15–20^ Only the studies based on the Korean Nation Health Insurance Service database^13^^,^^14^^,^^23^ and the AFFIRM trial^21^^,^^22^ followed up patients for >2 years. There was no significant difference between the results of studies with longer and shorter follow-up. However, there was no indication that studies had tested that the risk reduction due to ABC adherence remained constant over time although they used models that assumed proportional hazards.

Atrial fibrillation was denoted differently, with some studies based on AF trial cohorts where patients had AF confirmed by >30 s AF in ECG or 24 h Holter,^15–18^ while others relied on an AF diagnosis recorded in their electronic health records.^13^^,^^14^^,^^23^ Seven studies^12–14^^,^^16^^,^^19^^,^^20^^,^^23^ included all available AF patients within their cohorts, while some only included patients who were already high risk of stroke^21^^,^^22^ with some requiring a CHA_2_DS_2_-VASc score ≥2^17^^,^^18^ or for patients to have a specific comorbidity, such as diabetes mellitus.^15^ Thus, in five of these studies,^15^^,^^17^^,^^18^^,^^21^^,^^22^ all patients were eligible for OAC (based on CHA_2_DS_2_-VASc score). Five studies reported on stroke incidence,^13^^,^^14^^,^^17^^,^^20^^,^^21^ eight on all-cause mortality,^13–17^^,^^20–22^ two on cardiovascular mortality,^20^^,^^21^ five on bleeding,^13^^,^^14^^,^^17^^,^^20^^,^^21^ one on dementia,^23^ and three on hospitalization.^17^^,^^21^^,^^22^ Composite outcomes considering combinations of these outcomes were considered in 10 studies.^13–22^

**Figure 1:**
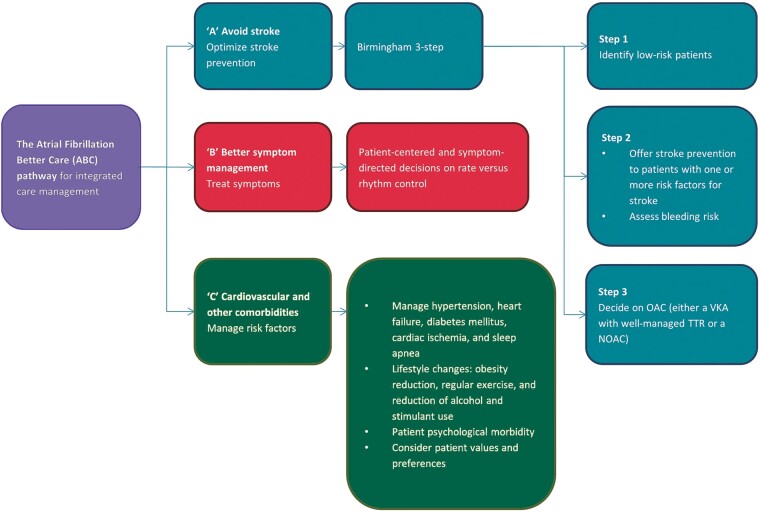
Flowchart of the steps in the ABC pathway. Adapted from Ref.^10^ ABC, Atrial Fibrillation Better Care; OAC, oral anticoagulant; NOAC, non-vitamin K antagonist oral anticoagulant; TTR, time in therapeutic range; VKA, vitamin K antagonist.

The different definitions for the individual components of the ABC pathway (*Figure 1*) used in the studies are shown in *Table 2*.

**Table 2:** Summary of criteria used by the included studies to define the A, B and C criteria of the Atrial fibrillation Better Care (ABC) pathway ^**10**^

	Components of the Atrial fibrillation Better Care (ABC) pathway and definitions utilized		
First author (year), country	Anticoagulation ‘A’	Better symptom management ‘B’	Cardiovascular and co-morbidity management ‘C’
Prospective			
Domek (2020)^15^	All high risk so OAC	EHRA classes I–II considered adherent	According to 2016 ESC AF guidelines^26^: *hypertension:* controlled <140/90 mm Hg, *HF:* ACEi/ARB or BB, *PAD:* statins or ACEi/ARB, *CAD:* statins or ACEi/ARB, *stroke/TIA:* statins
Gumprecht (2020)^16^	CHA_2_DS_2_-VASc for men/women 0/1: no OAC, 1+/2+: OAC	EHRA classes I–II considered adherent	According to 2016 ESC AF guidelines^26^: *Hypertension:* controlled <140/90 mm Hg, *HF:* ACEi or ARB along with BB, digoxin, and diuretic, *PAD:* statins or ACEi/ARB, *CAD:* ACEi or ARB along with BB, aspirin or clopidogrel, and LL drugs, *stroke/TIA:* withdraw OAC for short period depending on stroke severity and consider switching OAC if stroke while on OAC, *diabetes:* diet, insulin therapy, oral antidiabetic drugs
Guo (2020) 1 year and extension^17^^,^^18^	CHA_2_DS_2_-VASc > 2/3 for men/women: OAC. If on warfarin: weekly INR until stable and then monthly. Mean TiTR of 65% defined as good control	Evaluated using EHRA classification	*Hypertension:* <140/85 mm Hg or ideally 130/80 mm Hg., *vascular disease:* statins, *educational materials:* hypertension, heart failure, acute coronary syndrome (ACS), valvular disease, self-care
Koziel (2020)^12^	CHA_2_DS_2_-VASc for men/women: 0/1 no OAC, 1+/2+: OAC. Antiplatelet therapy should not be used concomitantly without clinical indications	EHRA classes II–IV considered adherent with rate or rhythm control strategy. EHRA class I not considered non-adherent but included in non-ABC adherent group	*Hypertension:* treated ≥140/90 mm Hg ACEi, AT1 receptor antagonist, CCB, BB, thiazide diuretic,^27^*HF:* ACEi, AT1 receptor antagonist, BB, thiazide diuretic, spironolactone, loop diuretic,^28^*CAD:* ACEi, AT1 receptor antagonist, CCB, BB, aspirin, statins, other LL drugs,^29^*diabetes:* lifestyle modifications, insulin therapy, oral antidiabetic drugs^30^
Retrospective—*post hoc*			
Proietti (2018, 2020)^21^^,^^22^	All patients on warfarin (cohort only includes those ≥65 years or with ≥1 risk factors for stroke). TiTR >70%	≤2 symptoms from: chest pain, diaphoresis, diuresis, dizziness, dyspnoea, oedema, fast heart rate, fatigue, orthopnoea, palpitations, panic, paroxysmal nocturnal dyspnoea, syncope, plus other symptoms	According to 2016 ESC guidelines^31^: *hypertension:* treated appropriately, <140/90 mm Hg, *HF:* ACEi + BB + diuretic, *PAD:* ACEi + LL drugs, *CAD:* ACEi + BB + LL drugs, *stroke/TIA:* LL drugs
Pastori (2019)^19^	CHA_2_DS_2_-VASc for men/women: 0/1 no OAC, 1/2 preferably OAC maybe aspirin, 2+/3 OAC. Warfarin used exclusively with TiTR > 65% over last year calculated by the Rosendaal method	EHRA classes I–II considered adherent	*Hypertension:* active management of ≥160/90 mm Hg with ARB, ACEi, BB, or mineralocorticoid receptor antagonist,^32^*HF:* ACEi or ARB along with BB along with further considerations,^33^*diabetes:* lifestyle modification, glucose control, insulin, and metformin first-line therapy for T1D and T2D, respectively^34^
Retrospective—registry or electronic health records			
Yoon (2019)^14^	Use of OACs in accordance with the guidelines with high adherence (prescription covering >80% of days)—does not reference which guidelines	<5 outpatient visits per year considered adherent	According to unspecified guidelines: *hypertension:* controlled <140/90 mm Hg, *HF:* ACEi or ARB along with BB, *MI:* ACEi or ARB along with BB and LL drugs, *PAD:* LL drugs, *diabetes:* oral anti-diabetics or insulin, *obesity:* BMI < 30 kg/m^2^
Proietti (2020) ESC-EHRA^20^	CHA_2_DS_2_-VASc for men/women: 0/1 no OAC, 1+/2+: OAC	EHRA classes I–II considered adherent	*Hypertension:* ≤140/90 mm Hg, *CAD:* ACEi, BB, and statins, *PAD:* statins, *previous stroke/TIA:* statins, *HF:* ACEi/ARB and BB, *diabetes:* insulin or oral antidiabetics
Yang (2020) frailty and dementia^13^^,^^23^	CHA_2_DS_2_-VASc for men/women: 0/1 no OAC, 1+/2+ OAC with prescription covering 80% of days	<5 visits per year considered adherent	According to 2016 ESC AF guidelines^26^: *hypertension:* controlled <140/90 mm Hg, *MI:* initially short period of triple therapy (OAC, aspirin, and clopidogrel) reducing to double (OAC and aspirin or clopidogrel), *HF:* ACEi or ARB along with BB, digoxin, and diuretic, *PAD:* statins or ACEi/ARB, *stroke/TIA:* withdraw OAC for short period depending on stroke severity and consider switching OAC if stroke while on OAC, *diabetes:* diet, insulin therapy, oral antidiabetic drugs, *obesity:* BMI < 30 kg/m^2^

ABC, Atrial Fibrillation Better Care; ACEi, angiotensin-converting-enzyme inhibitors; AF, atrial fibrillation; ARB, angiotensin receptor blockers; BB, beta-blocker; BMI, body mass index; CAD, coronary artery disease; ESC, European Society of Cardiology; EHRA, European Heart Rhythm Association; HF, heart failure; MI, myocardial infarction; OAC, oral anticoagulant; PAD, peripheral artery disease; TIA, transient ischaemic attack; TiTR, time in therapeutic range.

### ‘A’—avoid stroke with oral anticoagulation

All studies required OAC prescription for patients to be based on stroke risk identified with the CHA_2_DS_2_-VASc score. The definition of a high risk of stroke varied between studies. To meet the criteria for the ‘A’ component, one study considered OAC optional for patients with a CHA_2_DS_2_-VASc of 1 or 2 for men or women,^19^ respectively, while others considered that OAC was required in these patients.^13^^,^^16^^,^^20^^,^^23^ Five studies only included patients that had a CHA_2_DS_2_-VASc score ≥1 or ≥2 for men or women, respectively, meaning that all patients were eligible for OAC.^15^^,^^17^^,^^18^^,^^21^^,^^22^

Each study defined OAC adherence using different criteria. For patients receiving warfarin or other vitamin K antagonists (VKAs), time in therapeutic range (TiTR) was utilized to indicate anticoagulation control by five papers.^17–19^^,^^21^^,^^22^ For three studies,^17–19^ the target TiTR was >65% and in two others^21^^,^^22^ the target was >70%. TiTR was not always available; alternatively, prescription days coverage >80%^13^^,^^14^^,^^23^ was used.

### ‘B’—better symptom management

Seven studies defined adherence to the ‘B’ criterion as symptom levels classified as European Heart Rhythm Association (EHRA) classes I–II.^12^^,^^15–20^ Studies using the AFFIRM trial data allowed ≤2 symptoms from their own list.^21^^,^^22^ The studies based on the Korea National Health Insurance Service database did not have data on symptoms, therefore the authors used the criteria of <5 outpatient visits per year as a proxy.^13^^,^^14^^,^^23^

### ‘C’—cardiovascular and co-morbidity management

Each study considered a different set of conditions when defining the ‘C’ criteria as shown in *Table 2*. All studies considered hypertension although it was defined in multiple ways. Nine studies required blood pressure (BP) to be controlled at <140/90 mm Hg^12–16^^,^^20–23^ although other cut-offs (e.g. 160/90^19^ or 140/85^17^^,^^18^) were used.^17–19^ Two studies looked for active treatment of hypertension with pharmacological treatment rather than BP control.^12^^,^^19^ Each study looked at a different selection of other conditions such as diabetes,^12–14^^,^^16^^,^^19^^,^^20^^,^^23^ heart failure,^12–23^ peripheral artery disease,^13–16^^,^^20–23^ and coronary artery disease^12^^,^^15^^,^^16^^,^^20–22^; these were considered based on drugs used for prevention and/or treatment. Body mass index with a cut-off of 30 kg/m^2^ was considered for obesity in three studies.^13^^,^^14^^,^^23^

There was a wide-range in the proportion of participants assessed as ABC adherent in the included studies (7.0–43.8%),^12^^,^^21^^,^^22^ as shown in *Table 3*. Mean age varied among studies depending on the inclusion criteria. In three studies, those who were ABC adherent were over 10 years younger^12–14^ than those who were not ABC adherent; conversely in another study, ABC-adherent patients were over 8 years older.^16^ In four studies a lower proportion of ABC adherent patients were women,^14^^,^^20–22^ while in two studies a higher proportion were women.^12^^,^^19^ Hypertension was more prevalent in ABC non-adherent patients, although this was dependent on definitions.

**Table 3:** Summary of baseline characteristics by ABC adherence status for the selected studies

First author (year)	Grouped and overall	*N* (%)	Age, mean ± SD or median (IQR)	Women (%)	Hypertension (%)	CHA_2_DS_2_-VASc, mean ± SD or median (IQR)
Prospective						
Domek (2020)^15^	ABC	86 (14.3%)	64.8 ± 10.8	44 (51.2%)	69 (80.2%)	3.60 ± 1.27
	Non-ABC	517 (85.7%)	63.2 ± 11.9	271 (52.4%)	421 (81.4%)	3.70 ± 1.63
	All	603	63.4 ± 11.8	315 (52.2%)	490 (81.3%)	3.69 ± 1.58
Gumprecht (2020)^16^	ABC	168 (8.3%)	64.5 ± 12.0	77 (45.7%)	117 (69.6%)	3.01 ± 1.53
	Non-ABC	1853 (91.7%)	56.0 ± 16.7	891 (48.1%)	948 (51.2%)	2.28 ± 1.79
	All	2021	56.7 ± 16.47	968 (47.9%)	1065 (52.7%)	2.34 ± 1.78
Guo (2020) 1 year^18^	mAFA	1646 (49.5%)	67.0 ± 15.0	625 (38.0%)	908 (55.2%)	3 (2–4)
	Usual Care	1678 (50.5%)	70.0 ± 12.0	637 (38.0%)	962 (57.3%)	3 (2–4)
	All	3324	Not reported	1262	1870 (56.3%)	Not reported
Guo (2020) Extension^17^	mAFA	1261 (51.0%)	67.8 ± 15.4	34.1%	797 (63.2%)	3 (2–4)
	Usual Care	1212 (49.0%)	70.1 ± 12.0	42.1%	776 (64.0%)	3 (2–4)
	All	2473	Not reported	Not reported	Not reported	Not reported
Koziel (2020)^12^	ABC	1013 (43.8%)	49 (41–57)	485 (47.9%)	898 (88.6%)	3.4 ± 1.8
	Non-ABC	1299 (56.2%)	64 (55–71)	557 (42.9%)	882 (67.9%)	3.4 ± 1.9
	All	2712	Not reported	Not reported	Not reported	Not reported
Retrospective—*post hoc*						
Proietti (2018, 2020)^21^^,^^22^	ABC	222 (7.0%)	70 (65–75)	60 (27.0%)	141 (63.5%)	3 (2–4)
	Non-ABC	2947 (93.0%)	70 (65–76)	1177 (39.9%)	2102 (71.3%)	2 (1–3)
	All	3169	70 (65–76)	1237 (39.0%)	2243 (70.8%)	3 (2–4)
Pastori (2019)^19^	ABC	198 (22.4%)	71.7 ± 9.0	48.2%	85.6%	2.56 ± 1.1
	Non-ABC	684 (77.6%)	73.5 ± 8.3	38.7%	89.3%	3.7 ± 1.5
	All	882	73.1 ± 8.5	40.8%	88.5%	3.50 ± 1.5
Retrospective—registry or electronic health records						
Yoon (2019)^14^	ABC	31 674 (15.5%)	52.9 ± 12.2	10 129 (32.0%)	5708 (18.0%)	0.91 ± 1.39
	Non-ABC	173 168 (84.5%)	64.9 ± 10.8	66 778 (38.6%)	139 411 (80.5%)	2.97 ± 1.80
	All	204 842	Not reported	Not reported	Not reported	Not reported
Proietti (2020) ESC-EHRA^20^	ABC	1996 (30.0%)	70 (61–76)	741 (37.1%)	1184 (59.7%)	2.68 ± 1.57; 3 (2–4)
	Non-ABC	4650 (70.0%)	69 (61–76)	1926 (41.4%)	2693 (58.5%)	3.07 ± 1.90; 3 (2–4)
	All	6646	Not reported	Not reported	Not reported	Not reported
Yang (2020) dementia^23^	ABC	45 994 (20.2%)	68.8 ± 10.2	18 016 (39.2%)	2425 (5.3%)	0 (0–1)
	Non-ABC	182 052 (79.8%)	69.7 ± 11.6	70 218 (38.6%)	117 688 (64.7%)	2 (1–3)
	All	228 026	Not reported	Not reported	Not reported	Not reported
Yang (2020) frailty^13^	ABC	49 533 (18.8%)	50 (41, 58)	39.4%	7.0%	0 (0–1)
	Non-ABC	213 454 (81.1%)	65 (56, 72)	38.6%	65.5%	2 (1–3)
	All	262 987	Not reported	Not reported	Not reported	Not reported

ABC, Atrial Fibrillation Better Care; AF, atrial fibrillation; EHRA, European Heart Rhythm Association; ESC, European Society of Cardiology; mAFA, mobile AF-App.

**Figure 2:**
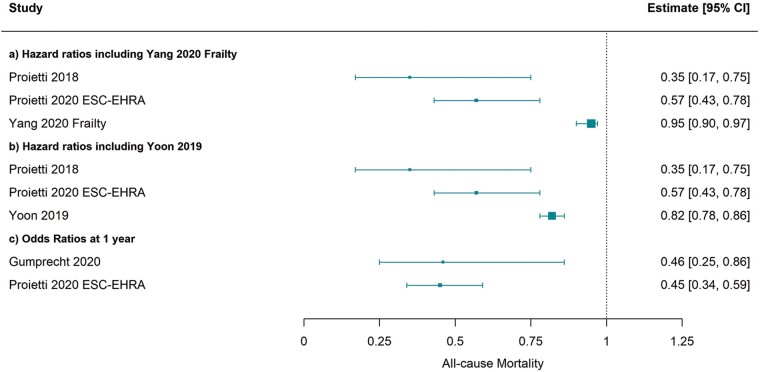
Forest plot depicting hazard ratios (*A* and *B*) and odds ratios (*C*) for ABC adherence vs. non-adherence for all-cause mortality. Yang and Yoon were analyses from subsets of the same dataset and were included separately in (*A*) and (*B*). ABC, Atrial Fibrillation Better Care; EHRA, European Heart Rhythm Association; ESC, European Society of Cardiology.

*Table 4* presents the outcomes in ABC-adherent vs. non-ABC adherent patients within each study. Each study adjusted for a different set of potential confounders, although age, sex, and diabetes status were adjusted for in eight of the studies.^13^^,^^14^^,^^17^^,^^18^^,^^20–23^ Due to different data availability, both Cox proportional hazards models and logistic regression were used to estimate the effect of ABC adherence on clinical outcomes. Hazard ratios (HRs) and odds ratios varied due to differing definitions but consistently reported that ABC pathway adherent care was beneficial for lowering mortality [*Figure 2, n* = 4 studies, HR 0.35 (95% confidence interval 0.17–0.75), HR 0.57 (0.43–0.78), HR 0.82 (0.78–0.86), and HR 0.93 (0.90–0.97)],^13^^,^^14^^,^^20^^,^^21^ cardiovascular mortality [Supplementary material online, *Figure S1*, n = 2 studies, HR 0.17 (0.04–0.70) and HR 0.52 (0.35–0.78)],^20^^,^^21^ major bleeding [Supplementary material online, *Figure S2*, n = 3 studies, HR 0.26 (0.08–0.81), HR 0.89 (0.84–0.94), and HR 0.99 (0.95–1.02)],^13^^,^^14^^,^^21^ stroke [*n* = 1 study, HR 0.86 (0.83–0.89)),^13^^,^^14^ myocardial infarction [*n* = 1 study, HR 0.76 (0.69–0.83)],^13^ hospitalization risk [*n* = 1 study, HR 0.65 (0.53–0.80)],^21^ and composites of these outcomes.

**Table 4:** Summary of the results and analysis by outcome among the included studies

First author (year)	Outcome	Adjustment variables	Adjusted hazard ratio/odds ratio
Prospective			
Domek (2020)^15^	All-cause mortality	AF type, renal dysfunction, dyslipidaemia, aspirin use, major bleeding	*ABC vs. non-ABC at 6 months:* OR 0.18 (0.04–0.75). *ABC vs. non-ABC at 1 year:* OR 0.29 (0.11–0.76). *AB vs. non-ABC at 1 year:* OR 0.73 (0.44–1.19). *AC vs. non-ABC at 1 year:* OR 0.72 (0.38–1.36). *BC vs. non-ABC at 1 year:* OR 0.53 (0.28–1.01)
	Composite: stroke/systemic embolism, all-cause mortality, CV hospitalization		*ABC vs. non-ABC at 6 months:* OR 0.54 (0.30–1.00). *ABC vs. non-ABC at 1 year:* OR 0.57 (0.33–0.97). *AB vs. non-ABC at 1 year:* OR 0.78 (0.54–1.12). *AC vs. non-ABC at 1 year:* OR 1.15 (0.74–1.77). *BC vs. non-ABC at 1 year:* OR 0.58 (0.37–0.91)
Gumprecht (2020)^16^	All-cause mortality	AF type, renal dysfunction, dyslipidaemia, aspirin use, major bleeding	*ABC vs. non-ABC at 6 months:* OR 0.31 (0.13–0.77). *ABC vs. non-ABC at 1 year:* OR 0.46 (0.25–0.86). *Standard care vs. AB vs. BC vs. AC at 1 year:* AB: OR 0.78 (0.58–1.06), AC: OR 0.95 (0.62–1.46), BC: OR 0.73 (0.47–1.13)
	Composite: ischaemic stroke or systemic embolism, all-cause mortality, and CV hospitalization		*ABC vs. non-ABC at 6 months:* OR 0.49 (0.31–0.79). *ABC vs. non-ABC at 1 year:* OR 0.53 (0.36–0.80). *Standard care vs. AB vs. BC vs. AC at 1 year:* AB: OR 0.75 (0.61–0.92), AC: OR 1.00 (0.74–1.36), BC: OR 0.68 (0.50–0.92)
Guo (2020) 1 year^18^	Composite: stroke/thromboembolism, all-cause mortality, and re-hospitalization	Age, sex, AF type, prior AF rhythm control, hypertension, diabetes, CAD, OSA, HF, hyperthyroidism, ischaemic stroke, dilated cardiomyopathy, HOCM	*mAFA vs. usual care:* overall: HR 0.39 (0.22–0.67), female: HR 0.48 (0.22–1.04, male: HR 0.34 (0.18–0.67), age <75 years: HR 0.17 (0.08–0.36), age ≥75 years: HR 0.63 (0.29–1.38), paroxysmal AF: HR 0.49 (0.25–0.94), persistent and permanent AF: HR 0.40 (0.17–0.94), CHA_2_DS_2_-VASc ≥2 in males, ≥3 in females: HR 0.57 (0.31–1.03), CHA_2_DS_2_-VASc 0–1 in males or 1–2 in females: HR 0.04 (0.01–0.27), HAS-BLED ≥3: HR 0.86 (0.35–2.16), HAS-BLED 0–2: HR 0.21 (0.12–0.37), hypertension: HR 0.52 (0.26–1.03), no hypertension: HR 0.11 (0.03–0.36), CAD: HR 0.53 (0.26–1.11), No CAD: HR 0.22 (0.11–0.44)
	Re-hospitalization		*mAFA vs. usual care:* overall: HR 0.32 (0.17–0.60), female: HR 0.27 (0.10–0.72, male: HR 0.31 (0.15–0.64), age <75 years: HR 0.17 (0.07–0.40), age ≥75 years: HR 0.46 (0.19–1.12), paroxysmal AF: HR 0.43 (0.19–0.94), persistent and permanent AF: HR 0.34 (0.13–0.86), CHA_2_DS_2_-VASc ≥2 in males, ≥3 in females: HR 0.41 (0.21–0.80), CHA_2_DS_2_-VASc 0–1 in males or 1–2 in females: HR 0.07 (0.01–0.55), HAS-BLED ≥3: HR 0.78 (0.24–2.56), HAS-BLED 0–2: HR 0.18 (0.09–0.38), hypertension: HR 0.33 (0.15–0.75), no hypertension: HR 0.17 (0.05–0.58), CAD: HR 0.45 (0.21–1.00), No CAD: HR 0.13 (0.04–0.38)
	Ischaemic stroke		*mAFA vs. usual care:* HR 1.31 (0.18–9.31)
	Other thromboembolism		*mAFA vs. usual care:* HR 1.02 (0.18–5.93)
	Extracranial bleeding		*mAFA vs. usual care:* HR 0.95 (0.54–1.66)
	Recurrent AF or AF symptoms		*mAFA vs. usual care:* HR 0.48 (0.29–0.79)
	Heart failure		*mAFA vs. usual care:* HR 0.99 (0.51–1.92)
	Acute coronary syndrome		*mAFA vs. usual care:* HR 0.21 (0.04–1.21)
	All-cause mortality		*mAFA vs. usual care:* HR 0.71 (0.26–1.91)
Guo (2020) extension^17^	Composite: stroke/thromboembolism, all-cause mortality, and re-hospitalization	Cluster effect, age, sex, CAD, diabetes mellitus, heart failure, PAD, pulmonary disease,^a^ dilated cardiomyopathy, prior ischaemic stroke, thromboembolism, intracranial bleeding, other bleeding, liver/renal dysfunction	*mAFA vs. usual care:* HR 0.18 (0.13–0.25)
	Ischaemic stroke		*mAFA vs. usual care:* HR 0.11 (0.05–0.27)
	Other thromboembolism		*mAFA vs. usual care:* HR 0.29 (0.09–0.94)
	Extracranial bleeding		*mAFA vs. usual care:* HR 0.37 (0.20–0.70)
	Recurrent AF or AF symptoms		*mAFA vs. usual care:* HR 0.33 (0.23–0.48)
	Heart failure		*mAFA vs. usual care:* HR 0.11 (0.24–0.66)
	Re-hospitalization		*mAFA vs. usual care:* HR 0.69 (0.49–0.97)
	All-cause mortality		*mAFA vs. usual care:* HR 0.94 (0.39–2.23)
Retrospective—*post hoc*			
Proietti (2018)^21^	All-cause mortality	Age, sex, diabetes, hepatic/renal disease, pulmonary disease, first AF episode, aspirin use	*ABC vs. non-ABC:* HR 0.35 (0.17–0.75). *Standard care vs. AB vs. BC vs. AC vs. ABC:* AB: HR 0.72 (0.48–1.08), BC: HR 0.64 (0.37–1.09), AC: HR 0.42 (0.24–0.76), ABC: HR 0.31 (0.15–0.67). *0 vs. 1 vs. 2 vs. 3 criteria fulfilled:* 1 criteria: HR 0.70 (0.55–0.90), 2 criteria: HR 0.49 (0.35–0.67), 3 criteria: HR 0.25 (0.12–0.55)
	Composite: stroke, major bleeding, CV mortality and first hospitalization		*ABC vs. non-ABC:* HR 0.35 (0.18–0.68). *Standard care vs. AB vs. BC vs. AC vs. ABC:* AB: HR 0.75 (0.53–1.07), BC: HR 0.68 (0.43–1.09), AC: HR 0.68 (0.43–1.09), ABC: HR 0.32 (0.16–0.62). *0 vs. 1 vs. 2 vs. 3 criteria fulfilled:* 1 criteria: HR 0.73 (0.59–0.91), 2 criteria: HR 0.54 (0.40–0.71), 3 criteria: HR 0.26 (0.13–0.52)
	Stroke		*ABC vs. non-ABC:* HR 0.90 (0.39–2.06)
	Major bleeding		*ABC vs. non-ABC:* HR 0.26 (0.08–0.81)
	CV mortality		*ABC vs. non-ABC:* HR 0.17 (0.04–0.70)
	First hospitalization		*ABC vs. non-ABC:* HR 0.65 (0.53–0.80)
	First CV hospitalization		*ABC vs. non-ABC:* HR 0.57 (0.43–0.77)
	Multiple hospitalizations		*ABC vs. non-ABC:* OR 0.38 (0.26–0.56)
	Total hospitalizations		*ABC vs. non-ABC:* beta −0.098
	Length of first hospital stay		*ABC vs. non-ABC:* beta −0.034
	Total length of all hospital stays		*ABC vs. non-ABC:* beta −0.061
Pastori (2019)^19^	Composite of CV events including: fatal/non-fatal ischaemic stroke, MI, TIA, cardiac revascularization (stent placement or coronary artery bypass), and cardiovascular mortality	Age ≥75 years, sex, paroxysmal AF	*ABC vs. non-ABC:* HR 0.44 (0.24–0.80)
Proietti (2020)^22^	Composite: all-cause hospitalization, all-cause mortality	Age, sex, first AF episode. *For multimorbidity subgroup:* aspirin use. *For polypharmacy subgroup:* diabetes, hepatic/renal disease, pulmonary disease. *For hospitalization subgroup:* diabetes, hepatic/renal disease, pulmonary disease, aspirin use	*Multimorbidity subgroup ABC vs. non-ABC:* HR 0.61 (0.44–0.85). *Polypharmacy subgroup ABC vs. non-ABC:* HR 0.68 (0.47–1.00). *Hospitalization subgroup ABC vs. non-ABC:* HR 0.59 (0.42–0.85). *Multimorbidity subgroup 0 vs. 1 vs. 2 vs. 3 criteria fulfilled:* 1 criteria: HR 0.73 (0.64–0.83), 2 criteria: HR 0.57 (0.49–0.82), 3 criteria: HR 0.47 (0.33–0.66). *Polypharmacy subgroup 0 vs. 1 vs. 2 vs. 3 criteria fulfilled:* 1 criteria: HR 0.70 (0.60–0.82), 2 criteria: HR 0.57 (0.47–0.69), 3 criteria: HR 0.51 (0.35–0.76). *Hospitalization subgroup 0 vs. 1 vs. 2 vs. 3 criteria fulfilled:* 1 criteria: HR 0.70 (0.60–0.81), 2 criteria: HR 0.64 (0.53–0.77), 3 criteria: HR 0.45 (0.31–0.65)
	All-cause mortality		*Multimorbidity subgroup ABC vs. non-ABC:* HR 0.23 (0.06–0.94). *Polypharmacy subgroup ABC vs. non-ABC:* HR 0.49 (0.16–1.54). *Hospitalization subgroup ABC vs. non-ABC:* HR 0.49 (0.18–1.33). *Multimorbidity subgroup 0 vs. 1 vs. 2 vs. 3 criteria fulfilled:* 1 criteria: HR 0.78 (0.59–1.02), 2 criteria: HR 0.50 (0.33–0.75), 3 criteria: HR 0.18 (0.05–0.75). *Polypharmacy subgroup 0 vs. 1 vs. 2 vs. 3 criteria fulfilled:* 1 criteria: HR 0.68 (0.48–0.94), 2 criteria: HR 0.51 (0.31–0.83), 3 criteria: HR 0.37 (0.12–1.18). *Hospitalization subgroup 0 vs. 1 vs. 2 vs. 3 criteria fulfilled:* 1 criteria: HR 0.61 (0.44–0.85), 2 criteria: HR 0.49 (0.31–0.76), 3 criteria: HR 0.36 (0.13–0.97)
	Hospitalization		*Multimorbidity subgroup ABC vs. non-ABC:* HR 0.62 (0.45–0.87). *Polypharmacy subgroup ABC vs. non-ABC:* HR 0.69 (0.46–1.01). *Hospitalization subgroup ABC vs. non-ABC:* HR 0.58 (0.40–0.84). *Multimorbidity subgroup 0 vs. 1 vs. 2 vs. 3 criteria fulfilled:* 1 criteria: HR 0.72 (0.63–0.82), 2 criteria: HR 0.57 (0.48–0.68), 3 criteria: HR 0.48 (0.34–0.67). *Polypharmacy subgroup 0 vs. 1 vs. 2 vs. 3 criteria fulfilled:* 1 criteria: HR 0.70 (0.60–0.82), 2 criteria: HR 0.57 (0.47–0.70), 3 criteria: HR 0.51 (0.35–0.76). *Hospitalization subgroup 0 vs. 1 vs. 2 vs. 3 criteria fulfilled:* 1 criteria: HR 0.70 (0.60–0.81), 2 criteria: HR 0.63 (0.53–0.76), 3 criteria: HR 0.44 (0.30–0.64)
	CV events		*Multimorbidity subgroup ABC vs. non-ABC:* HR 0.54 (0.35–0.84). *Polypharmacy subgroup ABC vs. non-ABC:* HR 0.67 (0.41–1.08). *Hospitalization subgroup ABC vs. non-ABC:* HR 0.48 (0.30–0.77). *Multimorbidity subgroup 0 vs. 1 vs. 2 vs. 3 criteria fulfilled:* 1 criteria: HR 0.71 (0.61–0.83), 2 criteria: HR 0.67 (0.55–0.81), 3 criteria: HR 0.43 (0.27–0.67). *Polypharmacy subgroup 0 vs. 1 vs. 2 vs. 3 criteria fulfilled:* 1 criteria: HR 0.61 (0.51–0.73), 2 criteria: HR 0.64 (0.51–0.79), 3 criteria: HR 0.49 (0.30–0.80). *Hospitalization subgroup 0 vs. 1 vs. 2 vs. 3 criteria fulfilled:* 1 criteria: HR 0.73 (0.61–0.87), 2 criteria: HR 0.75 (0.60–0.92), 3 criteria: HR 0.39 (0.24–0.63)
	Any event		*Multimorbidity subgroup ABC vs. non-ABC:* HR 0.60 (0.43–0.84). *Polypharmacy subgroup ABC vs. non-ABC:* HR 0.68 (0.46–0.99). *Hospitalization subgroup ABC vs. non-ABC:* HR 0.59 (0.41–0.84). *Multimorbidity subgroup 0 vs. 1 vs. 2 vs. 3 criteria fulfilled:* 1 criteria: HR 0.73 (0.64–0.83), 2 criteria: HR 0.59 (0.50–0.69), 3 criteria: HR 0.47 (0.33–0.65). *Polypharmacy subgroup 0 vs. 1 vs. 2 vs. 3 criteria fulfilled:* 1 criteria: HR 0.71 (0.61–0.82), 2 criteria: HR 0.58 (0.47–0.70), 3 criteria: HR 0.51 (0.34–0.75). *Hospitalization subgroup 0 vs. 1 vs. 2 vs. 3 criteria fulfilled:* 1 criteria: HR 0.69 (0.60–0.80), 2 criteria: HR 0.66 (0.55–0.79), 3 criteria: HR 0.45 (0.45–0.64)
Retrospective—registry or electronic health records			
Proietti (2020) ESC-EHRA^20^	Composite: thromboembolism, acute coronary syndrome, CV mortality	Type of AF, CHA_2_DS_2_-VASc score factors	*ABC vs. non-ABC at 1 year:* OR 0.48 (0.37–0.62)
	Stroke		*ABC vs. non-ABC at 1 year:* OR 0.78 (0.40–1.50)
	Any thromboembolism		*ABC vs. non-ABC at 1 year:* OR 0.60 (0.36–1.02)
	CV mortality		*ABC vs. non-ABC at 1 year:* OR 0.38 (0.27–0.54)
	All-cause mortality		*ABC vs. non-ABC at 1 year:* OR 0.45 (0.34–0.59)
	Acute coronary syndrome		*ABC vs. non-ABC at 1 year:* OR 0.68 (0.42–1.10)
	Any readmission		*ABC vs. non-ABC at 1 year:* OR 0.80 (0.71–0.91)
	Any AF readmission		*ABC vs. non-ABC at 1 year:* OR 0.86 (0.72–1.02)
	Any CV readmission		*ABC vs. non-ABC at 1 year:* OR 0.81 (0.71–0.93)
	Composite: thromboembolism, acute coronary syndrome, CV mortality		*ABC vs. non-ABC:* HR 0.59 (0.44–0.79). *0 vs. 1 vs. 2 vs. 3 criteria fulfilled:* 1 criteria: HR 0.68 (0.44–1.10), 2 criteria: HR 0.46 (0.29–0.74), 3 criteria: HR 0.31 (0.19–0.52)
	CV mortality		*ABC vs. non-ABC:* HR 0.52 (0.35–0.78). *0 vs. 1 vs. 2 vs. 3 criteria fulfilled:* 1 criteria: HR 0.60 (0.33–0.94), 2 criteria: HR 0.40 (0.24–0.66), 3 criteria: HR 0.25 (0.14–0.45)
	All-cause mortality		*ABC vs. non-ABC:* HR 0.57 (0.43–0.78). *0 vs. 1 vs. 2 vs. 3 criteria fulfilled:* 1 criteria: HR 0.69 (0.42–1.14), 2 criteria: HR 0.47 (0.29–0.76), 3 criteria: HR 0.32 (0.18–0.54)
	Haemorrhagic events	Type of AF, HAS-BLED score factors	*ABC vs. non-ABC at 1 year:* OR 0.78 (0.40–1.50)
	Intracranial haemorrhage	Type of AF, HAS-BLED score factors, sex	*ABC vs. non-ABC at 1 year:* OR 0.64 (0.18–2.27)
Yoon (2019)^14^	All-cause mortality	Age, sex, HF, hypertension, diabetes, previous ischaemic stroke/TIA	*ABC vs. non-ABC:* HR 0.82 (0.78–0.86). *Number of ABC criteria fulfilled with 0 baseline:* 1 criteria: HR 0.91 (0.88–0.94), 2 criteria: HR 0.86 (0.84–0.89), 3 criteria: HR 0.80 (0.77–0.84)
	Composite: mortality, ischaemic stroke, major bleeding, myocardial infarction		*ABC vs. non-ABC:* HR 0.86 (0.83–0.89). *Number of ABC criteria fulfilled with 0 baseline:* 1 criteria: HR 0.73 (0.70–0.75), 2 criteria: HR 0.63 (0.60–0.65), 3 criteria: HR 0.57 (0.53–0.60)
	Ischaemic stroke		*ABC vs. non-ABC:* HR 0.86 (0.82–0.91)
	Major bleeding		*ABC vs. non-ABC:* HR 0.89 (0.84–0.94)
	Myocardial infarction		*ABC vs. non-ABC:* HR 0.82 (0.72–0.90)
Yang (2020) dementia^23^	Dementia	Age, sex, HF, hypertension, diabetes mellitus, previous MI, PAD, economic status, CHA_2_DS_2_-VASc, HAS-BLED	*ABC vs. non-ABC:* overall: HR 0.80 (0.73–0.87), female: HR 0.75 (0.66–0.86), male: HR 0.84 (0.74–0.95), non-heart failure: HR 0.84 (0.76–0.93), heart failure: HR 0.63 (0.45–0.87), non-hypertension: HR 0.87 (0.77–0.97), hypertension: HR 0.93 (0.86–1.01), non-diabetes mellitus: HR 0.83 (0.75–0.91), diabetes mellitus: HR 0.62 (0.45–0.86), CHA_2_DS_2_-VASc 0–1: HR 1.06 (0.90–1.24), CHA_2_DS_2_-VASc ≥2: HR 0.80 (0.69–0.93), non-AF RFCA: HR 0.79 (0.72–0.87), AF RFCA: HR 1.40 (0.51–3.83), age ≥ 70: HR 0.82 (0.69–0.98), age 60–70: HR 0.93 (0.81–1.08), age 50–60: HR 1.05 (0.84–1.30), age <50: HR 0.94 (0.58–1.54)
	Alzheimer’s dementia		*ABC vs. non-ABC:* HR 0.79 (0.71–0.88)
	Vascular dementia		*ABC vs. non-ABC:* HR 0.76 (0.59–0.98)
Yang (2020) frailty^13^	All-cause mortality		*ABC vs. non-ABC:* overall: HR 0.93 (0.90–0.97), low frailty: HR 0.95 (0.91–0.99), intermediate frailty: HR 0.89 (0.82–0.97), high frailty: HR 0.74 (0.56–0.97)
	Ischaemic stroke		*ABC vs. non-ABC:* overall: HR 0.86 (0.82–0.91), low frailty: HR 0.88 (0.83–0.93), intermediate frailty: HR 0.75 (0.62–0.92), high frailty: HR 1.03 (0.72–1.49)
	Heart failure admission		*ABC vs. non-ABC:* overall: HR 0.84 (0.79–0.89), low frailty: HR 0.84 (0.79–0.89), intermediate frailty: HR 0.81 (0.68–0.95), high frailty: HR 0.89 (0.61–1.56)
	Acute myocardial infarction		*ABC vs. non-ABC:* overall: HR 0.76 (0.69–0.83), low frailty: HR 0.77 (0.69–0.85), intermediate frailty: HR 0.72 (0.56–0.94), high frailty: HR 0.69 (0.32–1.47)
	Major bleeding		*ABC vs. non-ABC:* overall: HR 0.99 (0.95–1.02), low frailty: HR 1.04 (0.96–1.09), intermediate frailty: HR 0.83 (0.75–0.91), high frailty: HR 0.72 (0.54–0.96)
	Composite: all-cause mortality, ischaemic stroke, heart failure admission, acute myocardial infarction, major bleeding		*ABC vs. non-ABC:* overall: HR 0.93 (0.90–0.97), low frailty: HR 0.95 (0.91–0.99), intermediate frailty: HR 0.89 (0.82–0.97), high frailty: HR 0.74 (0.56–0.97)

ABC, Atrial Fibrillation Better Care; AF, atrial fibrillation; CAD, coronary artery disease; CV, cardiovascular; ESC, European Society of Cardiology; EHRA, European Heart Rhythm Association; HOCM, hypertrophic cardiomyopathy; HR, hazard ratio; mAFA, mobile AF-App; MI, myocardial infarction; OR, odds ratio; OSA, obstructive sleep apnoea; PAD, peripheral artery disease; RFCA, radio frequency catheter ablation; TIA, transient ischaemic attack.

a Pulmonary disease includes chronic obstructive pulmonary disease, obstructive sleep apnoea syndrome, and pulmonary hypertension.

Four studies examined how the number of ABC criteria fulfilled impacted on the outcomes.^14^^,^^20–22^ The risk of mortality was reduced by meeting one [*n* = 3 studies, HR 0.70 (0.55–0.90), HR 0.69 (0.42–1.14), and HR 0.91 (0.88–0.94)], two [*n* = 3 studies, HR 0.49 (0.35–0.67), HR 0.47 (0.29–0.76), and HR 0.86 (0.84–0.89)], and three [*n* = 3 studies, HR 0.25 (0.12–0.55), HR 0.32 (0.18–0.54), and HR 0.80 (0.77–0.84)] ABC criteria compared with meeting no ABC criteria.^14^^,^^20^^,^^21^ There was also a risk reduction for cardiovascular mortality^20^ and composite outcomes.^14^^,^^20–22^ There was a consistent dose–response effect with more ABC-adherent criteria fulfilled translating into a lower risk for all outcomes.^14^^,^^20–22^

## Discussion

All nine studies that examined the risk of adverse outcomes among patients adherent to the ABC pathway reported a significant risk reduction of adverse events, with only one study showing a non-significant result for major bleeding.^13^ The risks of stroke, mortality, myocardial infarction, hospitalization, and composites of these outcomes have all been shown to be lower in patient’s adherent to the ABC pathway. None of the studies suggested that there was any negative effect of being adherent to the ABC pathway.

The significant positive effect of ABC pathway adherence was robust amongst the different datasets. However, there was a relatively large variation in the strength of the risk reduction (e.g. HRs ranged from 0.35 to 0.93 for mortality), reflecting the differences between the datasets, and criteria used to denote A, B, and C adherence which may result in differences in the degree of risk reduction. Several factors could be driving variation, for example, some of the studies only included patients with other stroke risk factors (e.g. older age or diabetes) and some studies used more robust definitions for ABC adherence. Seven of the included studies conducted a retrospective analysis of pre-existing datasets.^13^^,^^14^^,^^19–23^ The various retrospective analyses led to variation between the studies examined within this review including differences in the inclusion/exclusion criteria, definitions of ABC-adherence employed and study design. Lack of appropriate data, such as TiTR, AF symptoms, and treatment data for each of the criteria of the ABC pathway included, led to some studies using less comprehensive definitions^13^^,^^14^^,^^23^ than others.^12^^,^^15–22^

Care is needed when defining the ‘A’, ‘B’, and ‘C’ criteria to be used in retrospective studies as there is also the potential for healthier patients to be selected rather than just those who have had ABC adherent management. Not all criteria can be modified quickly after AF diagnosis and some require patient involvement, such as adherence to prescriptions, increasing TiTR, and reducing risk factors such as obesity.

All studies only examined if the patient’s care was adherent to the ABC pathway at baseline. However, risk factors have the potential to change over time,^24^ especially in patients that were newly diagnosed with AF at baseline. In studies with longer follow-up, changes from baseline are more likely. There was a large variation in follow-up length in the studies in this review, although all but two datasets had follow-up ≤2 years.^13^^,^^14^^,^^21–23^ Although all studies adjusted for the patient’s age when analysing the risk of adverse outcomes in patients adherent and non-adherent to the ABC pathway, only one stratified the results by different age groups.^23^ The results of this study suggested that there may be a greater risk reduction in older patients, but the study lacked power for this analysis.

Wagner *et al.*^25^ first purported the idea of integrated care for chronic diseases in 1996. The key to integrated care is engaging the patient in the decision-making process and management of their condition. Also crucial is involving a multidisciplinary team from specialists to carers in the success of AF management. These strategies aim to improve treatment adherence, reduce perceived treatment burden and provide better outcomes for the patient.

While some of the individual components that comprise the ABC pathway have previously been included in guidelines,^26^ the ABC pathway has recently been incorporated into the 2020 ESC guidelines for the management of AF,^3^ bringing these together in an easy to follow structure. This review adds to the evidence supporting the inclusion of the ABC pathway in AF guidelines and implementation in practice to improve patient outcomes. The heterogeneity of the retrospective cohorts and the ABC pathway assessments based on available data and outcomes are intrinsic to the particular studies; this could be avoided by prospective studies. The mAFA-II cluster randomized trial compared usual care against app-based mobile health (mHealth) intervention based on the ABC pathway^18^ and showed a risk reduction for those using the app-based care of 61% for a composite outcome of stroke/thromboembolism, all-cause mortality, and re-hospitalization and a risk reduction of 68% for re-hospitalization.

The long-term mAFA-II cohort showed high adherence and persistence of use, and maintenance of improved clinical outcomes with ABC pathway adherent management.^17^

### Strengths and limitations

This review has summarized all available studies that have examined the impact of ABC adherent vs. non-ABC adherent treatment in AF patients, showing a consistent clinically significant reduction in the risk of adverse outcomes for patients whose treatment is adherent to the ABC pathway. However, variation between the studies included in this review raises questions over the precise magnitude of the benefit of adherence to the ABC pathway in a general AF population using ideal definitions of ABC adherence. This variation in definitions and criteria included also precluded any attempts to combine the results of individual studies in a meta-analysis.

## Conclusion

All studies consistently showed statistically significant reductions in the risk of stroke, myocardial infarction, and mortality among those with treatment adherent to the ABC pathway. The ABC pathway provides a simple decision-making framework to enable consistent equitable care from clinicians in both primary and secondary/tertiary care. Further research examining the impact of ABC pathway implementation in prospective cohorts where consistent inclusion criteria and definitions of ‘A’, ‘B’, and ‘C’ adherent care can be used is needed.

## Supplementary material

Supplementary material is available at *Europace* online.

**Conflict of interest:** D.S. and R.K.-D. declared no conflict of interest. S.L.H. received investigator-initiated funding from Bristol-Myers Squibb. G.Y.H.L. is a consultant for Bayer/Janssen, BMS/Pfizer, Boehringer Ingelheim, Verseon, and Daiichi-Sankyo; speaker for BMS/Pfizer, Boehringer Ingelheim, and Daiichi-Sankyo; and no fees are directly received personally. D.A.L. received investigator-initiated educational grants from Bristol-Myers Squibb (BMS), has been a speaker for Boehringer Ingeheim and BMS/Pfizer, and has consulted for BMS, Bayer, Boehringer Ingelheim, and Daiichi-Sankyo. 

## Supplementary Material

euab092_supplementary_data
