# Supplementary material for: The Atrial Fibrillation Better Care pathway for managing atrial fibrillation: a review
Source: Europace. 2021 Jun 14;23(10):1511–27. doi: 10.1093/europace/euab092 (PMC8502499; doi:10.1093/europace/euab092)
Supplement: euab092_supplementary_data [file euab092_supplementary_data.docx]

0

0.25

0.5

0.75

1

1.25

Cardiovascular Mortality

Proietti 2020 ESC-EHRA

Proietti 2018

Proietti 2020 ESC-EHRA

0.52 [0.35, 0.78]

0.17 [0.04, 0.70]

0.38 [0.27, 0.54]

**Study**

**Estimate [95% CI]**

**Hazard ratios**

**Odds ratio at 1 year**

**Supplementary Figure 1:** Forest plot depicting the hazard ratios and odds ratios (95% CI) for ABC adherence vs. non-adherence for cardiovascular mortality.

Abbreviations: CI, confidence interval; EHRA, European Heart Rhythm Association; ESC, European Society of Cardiology

0

0.25

0.5

0.75

1

1.25

Major bleeding

Yang 2020 Frailty

Proietti 2018

Yoon 2019

Proietti 2018

0.99 [0.95, 1.02]

0.26 [0.08, 0.81]

0.89 [0.84, 0.94]

0.26 [0.08, 0.81]

**Study**

**Estimate [95% CI]**

**a) Hazard ratios including Yang 2020 Frailty**

**b) Hazard ratios including Yoon 2019**

**Supplementary Figure 2:** Forest plot depicting the hazard ratios and 95% CI for ABC adherence vs. non-adherence for major bleeding.

Yang 2020 and Yoon 2019 were analyses from subsets of the same dataset and were included separately in a) and b).
